# Intestinal Acid Sphingomyelinase Protects From Severe Pathogen-Driven Colitis

**DOI:** 10.3389/fimmu.2019.01386

**Published:** 2019-06-19

**Authors:** Jana Meiners, Vittoria Palmieri, Robert Klopfleisch, Jana-Fabienne Ebel, Lukasz Japtok, Fabian Schumacher, Ayan Mohamud Yusuf, Katrin A. Becker, Julia Zöller, Matthias Hose, Burkhard Kleuser, Dirk M. Hermann, Richard N. Kolesnick, Jan Buer, Wiebke Hansen, Astrid M. Westendorf

**Affiliations:** ^1^Institute of Medical Microbiology, University Hospital Essen, University of Duisburg-Essen, Essen, Germany; ^2^Institute of Veterinary Pathology, Free University of Berlin, Berlin, Germany; ^3^Department of Toxicology, Institute of Nutritional Science, University of Potsdam, Potsdam, Germany; ^4^Department of Molecular Biology, University of Duisburg-Essen, Essen, Germany; ^5^Department of Neurology, University Hospital Essen, University of Duisburg-Essen, Essen, Germany; ^6^Laboratory of Signal Transduction, Memorial Sloan-Kettering Cancer Center, New York, NY, United States

**Keywords:** *Citrobacter rodentium*, colitis, acid sphingomyelinase, amitriptyline, T_**h**_1, T_**h**_17

## Abstract

Inflammatory diseases of the gastrointestinal tract are emerging as a global problem with increased evidence and prevalence in numerous countries. A dysregulated sphingolipid metabolism occurs in patients with ulcerative colitis and is discussed to contribute to its pathogenesis. In the present study, we determined the impact of acid sphingomyelinase (Asm), which catalyzes the hydrolysis of sphingomyelin to ceramide, on the course of *Citrobacter (C.) rodentium*-driven colitis. *C. rodentium* is an enteric pathogen and induces colonic inflammation very similar to the pathology in patients with ulcerative colitis. We found that mice with Asm deficiency or Asm inhibition were strongly susceptible to *C. rodentium* infection. These mice showed increased levels of *C. rodentium* in the feces and were prone to bacterial spreading to the systemic organs. In addition, mice lacking Asm activity showed an uncontrolled inflammatory T_h_1 and T_h_17 response, which was accompanied by a stronger colonic pathology compared to infected wild type mice. These findings identified Asm as an essential regulator of mucosal immunity to the enteric pathogen *C. rodentium*.

## Introduction

Inflammatory bowel diseases (IBD), such as ulcerative colitis and Crohn's disease, are characterized by chronic, relapsing inflammatory conditions, resulting from a dysregulation of the mucosal immune system in the gastrointestinal tract (1). The exact mechanism underlying the pathogenesis of IBD is unknown; however, it is widely accepted that immunological abnormalities, genetic and environmental factors, as well as infections are important determinants of IBD (2).

Sphingolipids are a family of metabolic lipids that are ubiquitous in cellular membranes and include a bioactive subset that regulates various cellular mechanisms and biologic processes such as cell survival, growth, differentiation, and apoptosis (3). Interestingly, sphingolipids are also essential structural components of intestinal membranes, providing protection and integrity to the intestinal mucosa and regulating intestinal absorption processes (4, 5). Studies using common acute and chronic epithelial injury colitis models have shown that bioactive sphingolipids, particularly ceramide and sphingosine-1-phosphate, are important regulators of inflammation in IBD (6–8). The acid sphingomyelinase (Asm) is a relevant enzyme in this context, as it catalyzes the hydrolysis of sphingomyelin to ceramide. Asm is ubiquitously expressed and activated by a range of cellular stresses, including inflammatory cytokines and pathogens (9). The importance of Asm for cell functions was first recognized in Niemann-Pick disease type A and B, a genetic disorder with a severe accumulation of sphingomyelin in many organs (10). Recent studies implicated that Asm activity is also strongly involved in inflammatory processes (11). For example, Asm inhibition was shown to suppress the lipopolysaccharide (LPS) mediated release of inflammatory cytokines and to protect against disease pathology in chemical induced colitis in mice (12, 13). Furthermore, blockade of Asm bioactivity limited the *in vitro* differentiation of T helper cells derived from healthy volunteers and patients with Crohn's disease (14). These results implicate Asm inhibition as an innovative and effective immunoregulatory strategy for the treatment of IBD (12, 13, 15).

Nevertheless, the etiology of IBD is diverse and influenced by numerous factors (2). In this context, several enteropathogens have been implicated in the development of IBD (16), although to date, a causative bacterial agent for IBD has not been identified. Thus, further studies are needed to clarify the function of Asm under infectious and non-infectious conditions, as broad immunosuppression can increase the risk of infectious complications (17).

In the present study, we determined the impact of Asm activity on the course of *Citrobacter (C.) rodentium* induced colitis. In contrast to the protective effect of Asm inhibition in common acute and chronic epithelial injury colitis models, Asm inhibition or Asm deficiency strongly enhanced the susceptibility to enteric *C. rodentium* infection. Mice lacking Asm activity showed higher colon pathology, were prone to bacterial dissemination to the systemic organs, and showed an uncontrolled inflammatory T_h_1 and T_h_17 response compared to infected wild type mice. These findings identified Asm as a critical regulator of mucosal immunity to the enteric pathogen *C. rodentium*.

## Materials and Methods

### Mice

C57BL/6 mice were purchased from ENVIGO, Netherlands. To inhibit acid sphingomyelinase activity, amitriptyline or imipramine was administered to C57BL/6 mice at 180 mg/l via drinking water for 14 days prior to bacterial challenge, and for further 10 days of infection. Acid sphingomyelinase-deficient (*Smpd1*^−/−^) mice (18) were bred at the animal facility of the University Hospital Essen. All animals used in this study were 8–12 week old male or female mice kept in the animal experimental unit of the University Hospital Essen in individually ventilated cages and pathogen free conditions.

### Asm Activity

The activity of Asm in colonic tissue was quantified using BODIPY-labeled sphingomyelin as a substrate. After colonic tissue was pulverized and lysed in 250 mM sodium acetate (pH 5) and 1% NP-40, 2 μg protein was incubated with 100 pmol BODIPY-labeled sphingomyelin (Thermo Fisher Scientific, Germany) for 1 h at 37°C in 250 mM sodium actetate (pH 5) and 0.1% NP-40. The reaction was terminated by the addition of chloroform:methanol (2:1, v:v) to extract the lipids. Subsequently, the lower phase containing lipids was collected, dried in a SpeedVac at 37°C, dissolved in 20 μl chloroform:methanol (2:1, v:v) and transferred onto a thin layer chromatography (TLC) plate. Product and uncleaved substrate were separated using chloroform:methanol (80:20, v/v). After separation, spots were imaged using a Typhoon FLA 9500 and quantified with ImageQuant software.

### Ceramide and Sphingomyelin Quantification

Ceramide and sphingomyelin concentrations were quantified by rapid resolution liquid chromatography/mass spectrometry. Short lipids were extracted from colon biopsies with C17-ceramide and C16-d31sphingomyelin as internal standards, after homogenization of colonic tissue. Subsequently, samples were analyzed by rapid-resolution liquid chromatography-MS/MS using a Q-TOF 6530 mass spectrometer (Agilent Technologies, Waldbronn, Germany) operating in the positive ESI mode. The subsequent quantification was performed using Mass Hunter Software, and the resulting sphingolipid quantities were normalized to the actual protein content of the homogenate.

### *C. rodentium* Infection Model

*C. rodentium* ICC169 strain was cultured overnight in Luria-Bertani (LB) medium at 37°C, centrifuged and washed with PBS. Mice were infected by oral gavage with ~2 × 10^9^ colony forming units (CFUs) of *C. rodentium*. After gavage, an aliquot of the bacteria was plated in serial dilutions on MacConkey agar. Bacterial numbers in the feces were determined at indicated time points after infection. Mice were analyzed at various time points after infection (d.p. infection).

### Intestinal Permeability-Assay

For *in vivo* analysis of the intestinal permeability fluorescein isothiocyanate-conjugated (FITC)-dextran beads have been used. Briefly, food and water were withdrawn for 2 h and mice were orally administrated with permeability tracer (60 mg/100 g body weight of FITC-labeled dextran, MW 4000; FD4, Sigma-Aldrich, St. Louis, USA). Serum was collected 4 h later and fluorescence intensity was determined (excitation, 492 nm; emission, 525 nm; BioTek). FITC-dextran concentrations were determined using a standard curve generated by serial dilution of FITC-dextran.

### Isolation of Splenocytes and Mesenteric Lymph Node Cells

Spleens were rinsed with an erythrocyte lysis buffer [containing 0.15 M NH_4_Cl, 10 mM KHCO_3_, and 0.5 M ethylenediaminetetraacetic acid (EDTA)], meshed through a 100-μm cell strainer, and washed with PBS containing 2 mM EDTA and 2% fetal calf serum (FCS). Mesenteric lymph nodes (mLN) were meshed through a 100-μm cell strainer and washed with PBS containing 2 mM EDTA and 2% FCS.

### Isolation of Lamina Propria Lymphocytes From the Colon

Lamina propria (LP) lymphocytes were isolated as described previously (19). In brief, colons were flushed with PBS, opened longitudinally, and cut into 1-cm pieces. Tissue pieces were washed twice in PBS containing 3 mM EDTA for 10 min at 37°C and twice in Roswell Park Memorial Institute (RPMI) medium containing 1% FCS, 1 mM EGTA, and 1.5 mM MgCl_2_ for 15 min at 37°C. Colon pieces were intensively vortexed, washed with phosphate-buffered saline (PBS), and digested in RPMI containing 20% FCS and 100 U/mL collagenase (*Clostridium histolyticum*; Sigma-Aldrich, St. Louis, MO) for 60 min at 37°C. Cell suspension was passed through a 40-μm cell strainer and washed with culture medium.

### Macroscopic and Histopathologic Assessment of Colitis

Colonic damage was assessed based on two main characteristics: colon length and colon weight. Colons were embedded in paraffin, and tissue sections (4 μm) were prepared for histological scoring in a blinded manner. The colon was divided into three equal portions (oral, middle, and rectal) and assessed for inflammatory cell infiltrates, epithelial damage, neutrophil infiltration, crypt abscesses, and crypt hyperplasia as describe before (20). Crypt heights were measured by micrometry; 30 measurements were taken in the distal colon for each mouse.

### Cytokine Detection

Cytokines in serum samples were quantified using a Procarta Cytokine assay kit, according to the manufacturer's guidelines. The assay was run on a Luminex 200 system and cytokine levels were quantified using the Luminex IS software (Luminex Corporation, Austin, TX).

### Antibodies and Flow Cytometry

Cells were stained with fluorochrome-labeled anti-mouse CD4 (RM4-5), CD11b (M1/70), F4/80 (BM8), FoxP3 (FJK-16s), IFNγ (XMG1.2), IL-17 (TC11-18H10.1), and I-A/I-E (M5/114.15.2) antibodies and analyzed by flow cytometry on an LSR II instrument using DIVA software (BD Biosciences).

### Statistical Analysis

Normality of data was tested using D'Agostino & Pearson normality test and Shapiro–Wilk normality test. Statistical analysis was performed using Student's *t-*test, one-way ANOVA or two-way ANOVA followed by Tukey's multiple comparisons test, Dunn's multiple comparisons test or Bonferroni's multiple comparisons test. *P*-values were set at a level of *p* < 0.05. Statistical analyzes were performed using GraphPad Prism software version 7.

### Ethics Statement

This study was carried out in accordance with the recommendations of the Society for Laboratory Animal Science (GV-SOLAS) and the European Health Law of the Federation of Laboratory Animal Science Associations (FELASA). The protocol was approved by the North Rhine-Westphalia State Agency for Nature, Environment and Consumer Protection (LANUF), Germany.

## Results

### Alterations of the Sphingolipid Profile During *C. rodentium* Infection

**Figure 1:**
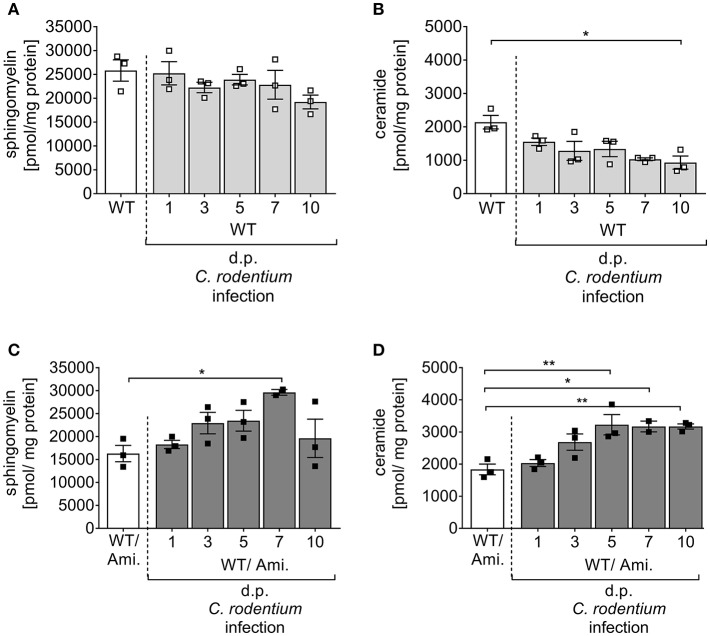
Alterations of sphingolipid concentrations during *C. rodentium* infection. C57BL/6 mice were either left untreated (WT) or pre-treated with 180 mg/l amitriptyline in drinking water 2 weeks prior to infection (WT/Ami) and during the course of infection. Mice were orally gavaged with PBS or with ~2 × 10^9^ colony forming units (CFUs) of *C. rodentium*. At indicated days post (dp) infection colons were excised. In rectal colonic tissue, the **(A,C)** sphingomyelin and **(B,D)** ceramide contents were analyzed using rapid resolution liquid chromatography/mass spectrometry. Data are presented as the concentration of ceramide and sphingomyelin in pmol/mg protein. All data are presented as mean ± SEM. Statistics were performed using the parametric one-way ANOVA test with Tukey's multiple comparison test (^*^p < 0.05; ^**^p < 0.01).

Sphingolipids have been identified as important players to control intestinal inflammation. There is increasing evidence that a dyregulaton of several sphingolipid molecules occurs along with IBD and contributes to the pathogenesis and maintenance of the disease (21). To analyze the impact of the sphingolipid metabolism on pathogen-driven intestinal inflammation, C57BL/6 wild type (WT) mice were infected via oral gavage with ~2 × 10^9^ CFUs *C. rodentium*, and the sphingomyelin and ceramide concentrations were determined in the colon at indicated time points post infection by mass spectrometry. Interestingly, sphingomyelin as well as ceramide concentrations in the colon decreased during the course of infection in comparison to non-infected WT mice (Figures 1A,B), suggesting an involvement of the sphingolipid metabolism in intestinal inflammation.

### Oral Amitriptyline Pre-treatment Inhibits the Colonic Asm Activity During *C. rodentium* Infection

Acid sphingomyelinase (Asm) metabolizes sphingomyelin into ceramide, and this enzyme can be pharmacologically inhibited by amitriptyline (Ami) (22, 23). Amitriptyline accumulates in the acid compartments of the lysosomes, interferes with the translocation of Asm to the outer leaflet of the membrane and inhibits the activation of Asm (9). As sphingomyelin and ceramide were downregulated during the course of *C. rodentium* infection, we tested if the inhibition of Asm protects from pathogen-driven intestinal inflammation. For the specific *in vivo* inhibition of Asm in the gastrointestinal tract, WT mice were provided with amitriptyline in the drinking water for 14 days before and during a 10 day course of infection (WT/Ami). To check for the inhibition of the Asm activity, colonic tissues of WT/Ami mice, infected and non-infected, were analyzed for their sphingolipid profile by mass spectroscopy during the course of infection. Although no differences in the sphingomyelin and ceramide concentration were discovered under homeostasis, we detected an accumulation of sphingomyelin and ceramide in the colon of infected WT/Ami mice with constant increase during the course of infection (Figures 1C,D). This data clearly demonstrated a modulation of the sphingolipid pathway in the colonic tissue by amitriptyline treatment via the drinking water.

### Asm Inhibition Increases the Susceptibility to *C. rodentium* Infection

**Figure 2:**
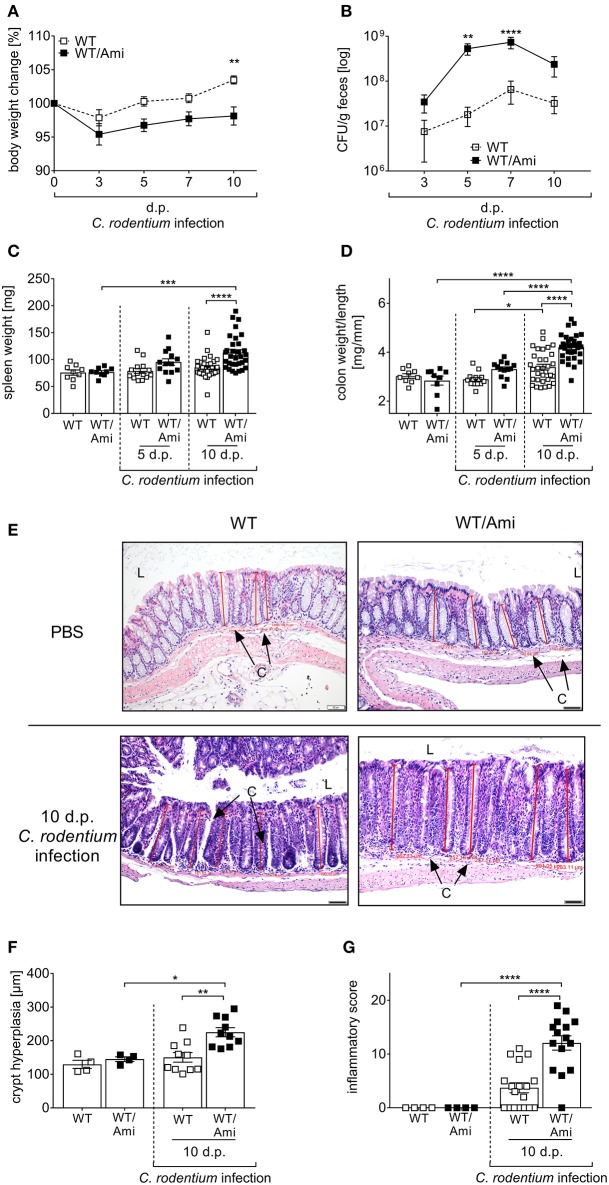
Amitriptyline pre-treatment increases the susceptibility to *C. rodentium* challenge. C57BL/6 mice were treated as described in Figure 1. **(A,B)** At indicated time points body weight and CFUs in feces in WT mice (white squares; *n* = 32–33) and WT/Ami mice (black squares; *n* = 32–35) were assessed. Statistics were performed using the Mann–Whitney test. **(C)** Spleen weight (*n* = 9–32) and **(D)** colon weight-to-length ratio were determined in uninfected, untreated and uninfected amitriptyline treated mice 5 and 10 dp *C. rodentium* infection (*n* = 9–32). **(E)** Representative H&E staining of colon sections from PBS or *C. rodentium* infected WT or WT/Ami mice 10 dp challenge. [Red lines indicate the crypt length. (L) lumen, (C) crypt. Length of scale bar is 50 μm]. **(F)** Measured crypt length in colons of uninfected WT or WT/Ami mice, and infected WT and WT/Ami 10 dp *C. rodentium* infection (*n* = 4–18). **(G)** Histopathology score of uninfected WT and WT/Ami mice, and WT and WT/Ami mice 10 dp *C. rodentium* infection (*n* = 4–18). All data are presented as mean ± SEM. Statistics were performed using the two-way ANOVA test followed by Tukey's multiple comparison test (^*^p < 0.05; ^**^p < 0.01; ^***^p < 0.001; ^****^p < 0.0001).

To further elucidate the physiological effect of Asm inhibition on the course of infection, we characterized the *C. rodentium* induced inflammatory response in WT and WT/Ami mice in detail. First, we determined the body weight loss of the mice as indicator for diarrhea. Surprisingly, *C. rodentium* infected WT/Ami mice lost significantly more body weight within 5 and 10 days post infection compared to infected wild type mice (Figure 2A). Determination of the bacterial burden in the feces showed that bacterial loads were similar in WT and WT/Ami infected mice on day 3 post infection with 10^7^ CFUs/g of feces. However, *C. rodentium* infected WT/Ami mice exhibited significantly higher bacterial numbers at day 5 and 7 post infection than did infected WT mice (Figure 2B), suggesting an impairment in the clearance of the pathogen. Interestingly, reduced bacterial eradication in WT/Ami mice was associated with exaggerated inflammation, characterized phenotypically by higher spleen weights (Figure 2C) and significantly higher colon weight-to-length ratios (Figure 2D) than in infected WT mice 10 days post infection. Well in line, histological analysis of the colon showed that *C. rodentium*-infected WT/Ami mice exhibited more severe crypt elongation and crypt hyperplasia (Figures 2E,F) and a higher inflammatory score compared to infected WT mice (Figure 2G). Of note, the same phenotype was obtained when infected mice were treated daily with amitriptyline via intraperitoneal injection (data not shown). These findings indicate that Asm activity is not only important for the eradication of *C. rodentium* but also for the control of infection-associated inflammation. Therefore, inhibition of Asm is not protective in pathogen-driven intestinal inflammation.

### Asm Provides Host Resistance to Bacterial Dissemination

**Figure 3:**
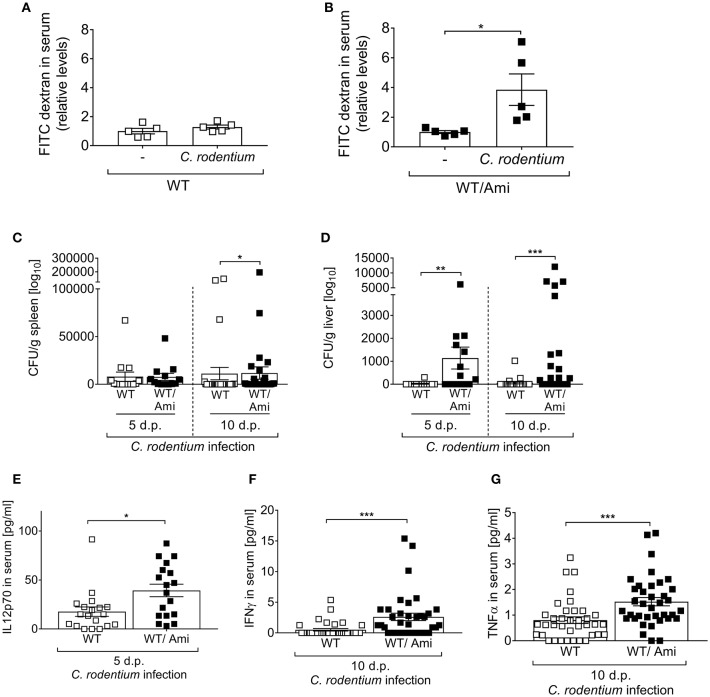
Enhanced systemic distribution of *C. rodentium* in mice pre-treated with amitriptyline. C57BL/6 mice were treated and infected as described in Figure 1. **(A,B)** Mice were orally administrated with FITC-labeled dextran beads. Serum was collected 4 h later, and fluorescence intensity was determined (*n* = 5). Serum FITC concentration in infected mice was normalized to serum FITC concentration of the respective control. **(C,D)** 5 and 10 dp *C. rodentium* infection spleen and liver were isolated and CFUs of *C. rodentium* were assessed (*n* = 13–27). **(E–G)** Concentration of the cytokines IL12p70, IFNγ, and TNFα in serum of WT and WT/Ami mice 10 dp *C. rodentium* infection were measured using Luminex technologies (*n* = 18–37). All data are presented as mean ± SEM. Statistics were performed using the Mann–Whitney test or Student's *t*-test (^*^p < 0.05; ^**^p < 0.01; ^***^p < 0.001).

A consequence of certain enteric bacterial infections is a breakdown of the intestinal barrier, allowing pathogen spreading from the intestine to the systemic organs of a host. Therefore, we tested whether Asm inhibition affects the intestinal permeability *in vivo*. Non-infected and infected WT and WT/Ami mice were orally gavaged with FITC-labeled dextran beads and 4 h later the intestinal permeability was assessed as relative concentration of serum FITC-dextran. Interestingly, the FITC concentrations in the serum of WT/Ami mice were significantly increased when compared to non-infected WT/Ami controls (Figure 3B). In contrast, only a slight but not significant increase in serum FITC levels was detected in infected WT mice compared to non-infected WT mice (Figure 3A).

These results led us to hypothesize that bacteria, once invaded into the gastrointestinal tract, are spread to the systemic organs of infected WT/Ami mice. Thus, we infected WT and WT/Ami mice via oral gavage with ~2 × 10^9^ CFUs *C. rodentium* per mouse, harvested the spleen and liver 5 and 10 days post-infection and analyzed the presence of viable bacteria. Well in line with the permeability assay, a higher bacterial burden was observed in the livers and spleens of infected WT/Ami mice compared to infected WT mice (Figures 3C,D).

The production of protective cytokines is a hallmark of immune responses being mounted toward the infection. To assess if the systemic distribution of *C. rodentium* in WT/Ami mice altered the level of cytokines that have been shown to orchestrate the immune response against *C. rodentium* infection (24), we infected WT and WT/Ami mice and measured the cytokine levels of IL-12p70, IFNγ, and TNFα in the sera. Of note, we found significantly elevated levels of IL12p70, INFγ, and TNFα in the sera of infected WT/Ami mice on day 10 post infection compared to infected WT mice (Figures 3E–G). Hence, Asm inhibition did not impair the production of protective pro-inflammatory cytokines. The high levels of certain pro-inflammatory cytokines in infected WT/Ami mice could be a consequence of the increased number of bacteria in their colon and liver tissues.

### Colonic Infiltration of Macrophages Is Not Impaired by Asm Inhibition

**Figure 4:**
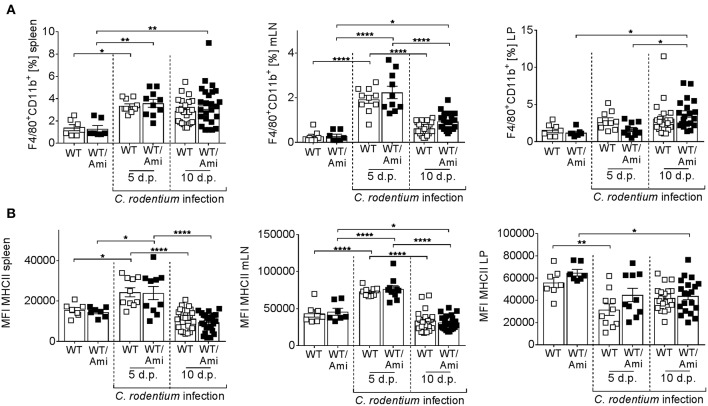
Infiltration of macrophages into colonic tissue is not altered in amitriptyline pre-treated mice after *C. rodentium* challenge. C57BL/6 mice were treated as described in Figure 2. Cells from the spleen, mesenteric lymph nodes (mLNs), and the lamina propria (LP) were isolated from uninfected WT and WT/Ami mice, 5 and 10 days post *C. rodentium* infection and analyzed for the frequency for macrophages by flow cytometry. **(A)** Percentages of macrophages from living cells are displayed (*n* = 7–25). **(B)** MFI of MHCII from macrophages is displayed (*n* = 7–25). All data are presented as mean ± SEM. Statistics were performed using the two-way ANOVA test with Tukey's multiple comparison test (^*^p < 0.05; ^**^p < 0.01; ^****^p < 0.0001).

Intestinal macrophages and macrophage-derived IL-12 are required for the initiation of adaptive immunity in response to *C. rodentium* (25). To investigate the role of Asm in mucosal immunity against *C. rodentium* infection, we measured the frequency of macrophages in the spleen, mesenteric lymph nodes, and the colonic lamina propria of WT and WT/Ami mice infected with *C. rodentium*. At day 5 and day 10 post infection the frequencies of macrophages were enhanced in the spleen and the LP, but no differences were found between infected WT and infected WT/Ami mice (Figure 4A). Furthermore, the expression of MHC II, which is essential for the activation of CD4^+^ T cells, was not impaired in infected WT/Ami mice compared to infected WT mice (Figure 4B). These data suggest that the function of macrophages to initiate adaptive immunity is independent of Asm during *C. rodentium* infection.

### Asm Inhibition Leads to an Uncontrolled Expansion of T_**h**_1 and T_**h**_17 Cells

**Figure 5:**
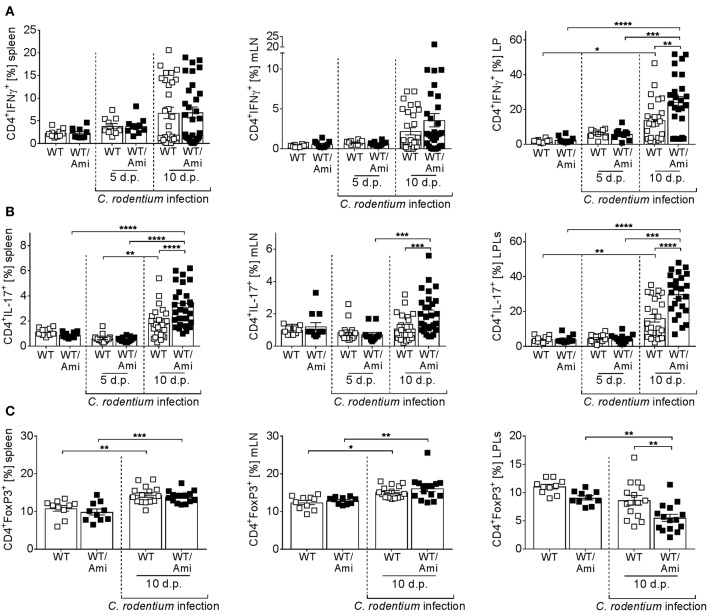
Enhanced proportions of T_h_1 and T_h_17 cells in colonic tissue of amitriptyline treated mice. C57BL/6 mice were treated as described in Figure 2. Single cells from spleen, mesenteric lymph nodes (mLNs), and lamina propria (LP) were prepared from uninfected WT or WT/Ami mice, 5 and 10 days post *C. rodentium* infection, and analyzed for **(A)** CD4^+^IFNγ^+^ cells (T_h_1), **(B)** CD4^+^IL-17^+^ cells (T_h_17), or **(C)** CD4^+^FoxP3^+^ cells (T_regs_) by flow cytometry. Percentages of T_h_1, T_h_17, and T_regs_ from CD4^+^ T cells are displayed. All data are presented as mean ± SEM. Statistics were performed using the two-way ANOVA test with Tukey's multiple comparison test (^*^p < 0.05; **p < 0.01; ***p ^<^ 0.001; ^****^p < 0.0001).

Infection with *C. rodentium* is associated with the induction of T_h_1 and T_h_17 adaptive immune responses (26–31). To measure the CD4^+^ T cell response during infection of WT and WT/Ami mice, splenocytes, mLN cells, and LP cells were restimulated *ex vivo* and analyzed for IFNγ and IL-17 production via flow cytometry. Comparable frequencies of T_h_1 and T_h_17 cells were detected in uninfected mice treated with or without amitriptyline. In contrast, *C. rodentium* infected mice exhibited enhanced frequencies of IFNγ- and IL-17-producing cells at day 10 post infection. Compared with infected WT controls, WT/Ami mice displayed an overall increase in activated T cells, with highest frequencies of T_h_1 and T_h_17 cells at day 10 post infection (Figures 5A,B).

Regulatory T cells (T_regs_) are important to counterbalance effector T cell responses and to protect from severe pathology (32). Recently, it was shown that deficiency of T_regs_ enhances the susceptibility to *C. rodentium* infection (32). Therefore, we assessed the frequencies of T_regs_ in the spleen, mLN, and LP of *C. rodentium* infected WT and WT/Ami mice. No differences were observed in the spleens and mLN of non-infected and infected WT mice compared to infected WT/Ami mice. Nonetheless, the percentage of CD4^+^Foxp3^+^ T_regs_ was significantly decreased in the LP of infected WT/Ami mice compared to infected WT mice (Figure 5C). In summary, Asm inhibition enhances the colonic frequencies of T_h_1 and T_h_17 cells in *C. rodentium* infected mice, which seems to be a consequence of increased numbers of bacteria in the colon and a disturbed counterbalanced induction of T_regs_.

### Asm Knock-Out Mice Are Strongly Susceptible to *C. rodentium* Infection

**Figure 6:**
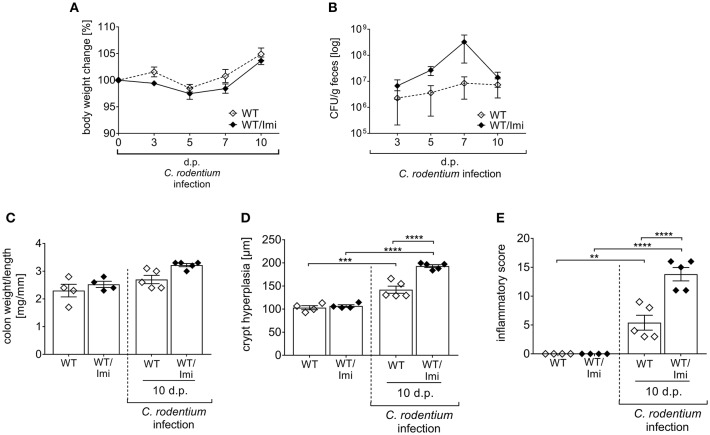
Imipramine pre-treatment increases the susceptibility to *C. rodentium* challenge. C57BL/6 mice were either left untreated (WT) or pre-treated with 180 mg/l imipramine in drinking water 2 weeks prior to infection (WT/Imi) and during the course of infection. Mice were orally gavaged with PBS or with ~2 × 10^9^ colony forming units (CFUs) of *C. rodentium*. **(A,B)** At indicated time points body weight and CFUs in feces in WT mice (white diamonds; *n* = 4–5) and WT/Imi mice (black diamonds; *n* = 4–5) were assessed. Statistics were performed using the Mann–Whitney test. **(C)** Colon weight-to-length ratio were determined in uninfected, untreated, and uninfected imipramine treated mice 10 dp *C. rodentium* infection (*n* = 4–5). Crypt hyperplasia **(D)** and inflammation score **(E)** of uninfected WT and WT/Imi mice, and WT and WT/Imi mice 10 dp *C. rodentium* infection (*n* = 4–5). All data are presented as mean ± SEM. Statistics were performed using the one-way ANOVA test followed by Tukey's multiple comparison test (^**^p < 0.01; ^***^p < 0.001; ^****^p < 0.0001).

Amitriptyline was initially introduced in order to treat major depressive disorders (33). However, nowadays the use of amitriptyline has expanded to numerous types of pain and other symptoms (34–38). In addition, anti-inflammatory and anti-microbial properties of the drug have been reported as well (39, 40). To exclude eventual drug unspecific effects, we used a second pharmacological Asm inhibitor, imipramine (Imi) within the same experimental set up. Well in line with the results from amitriptyline treated mice, imipramine treated mice are more susceptible to *C. rodentium* induced colitis, as shown by increased bacterial burden in the feces, enhanced colon-weight-to-length ratios and more severe pathology compared to infected WT mice (Figures 6A–E).

**Figure 7:**
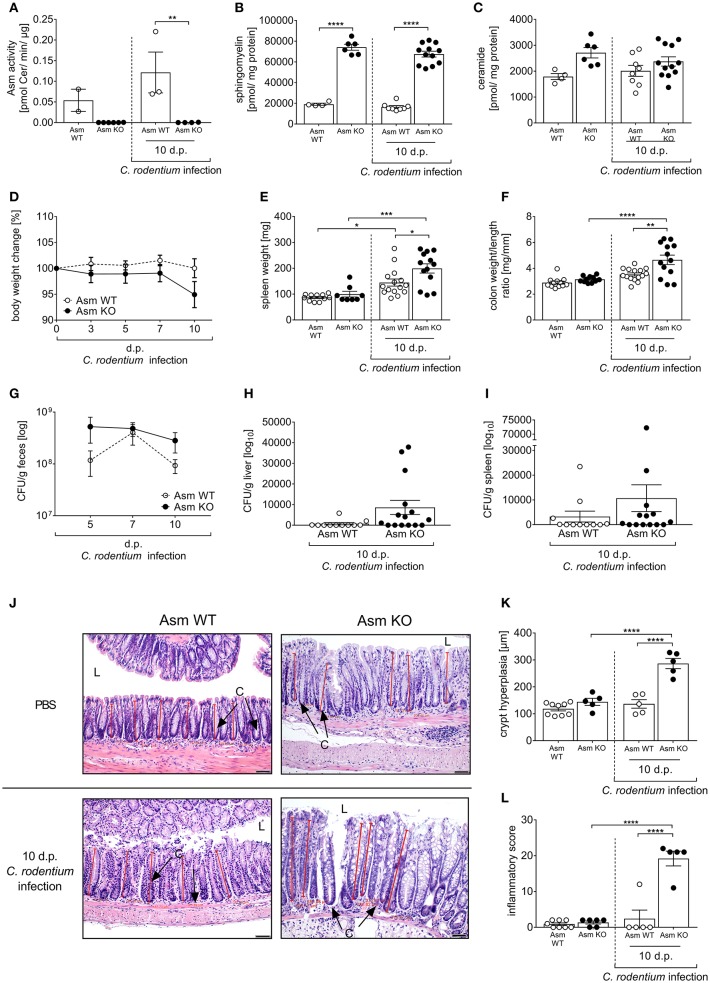
Deficiency of acid sphingomyelinase (Asm) increases susceptibility to *C. rodentium* infection. Asm wildtype (Asm WT) and Asm knock-out (Asm KO) mice were orally gavaged with PBS or ~2 × 10^9^ colony forming units (CFUs) of *C. rodentium*. Colons were excised from uninfected Asm WT and Asm KO mice and 10 dp infection. **(A)** Asm activity was analyzed in rectal colonic tissue (*n* = 2–6) by rapid resolution liquid chromatography/mass spectrometry. **(B)** Sphingomyelin (*n* = 4–12) **(C)** and ceramide (*n* = 4–12) concentrations are displayed. **(D)** At indicated time points body weight was assessed in Asm WT mice (*n* = 16–17) and Asm KO mice (*n* = 14–20). Statistics were performed using the Mann–Whitney test or the Student's *t*-test. **(E)** Spleen weight (*n* = 8–15) and **(F)** colon weight-to-length ratio were determined in uninfected, untreated, and uninfected amitriptyline treated mice 5 and 10 dp *C. rodentium* infection (*n* = 11–15). **(G–I)** At indicated time points CFUs in feces, liver, and spleen of Asm WT mice (*n* = 11–12) and Asm KO mice (*n* = 14–15) were assessed. Statistics were performed using the Mann–Whitney test or the Student's *t*-test. **(J)** Representative H&E staining of colon sections from PBS or *C. rodentium* infected Asm WT or Asm KO mice 10 dp infection. [Red lines indicate the crypt length. (*L*) lumen, (*C*) crypt. Length of scale bar−50 μm]. **(K)** Crypt length in colons of uninfected Asm WT or Asm KO mice, and infected Asm WT and Asm KO 10 dp *C. rodentium* infection (*n* = 5–9). **(L)** Histopathology score of the colon of uninfected Asm WT and Asm KO mice, and Asm WT and Asm KO mice 10 dp *C. rodentium* infection (*n* = 5–8). All data are presented as mean ± SEM. Statistics were performed using the two-way ANOVA test followed by Tukey's multiple comparison test (^*^p < 0.05; ^**^p < 0.01; ^***^p < 0.001; ^****^p < 0.0001).

To prove that the effect in amitriptyline and imipramine treated animals on *C. rodentium* induced inflammation is specifically due to the inhibition of Asm, we repeated the infection experiments in Asm wild type (Asm WT) and Asm knock-out (Asm KO) mice. First, we confirmed the specific depletion of Asm in Asm KO mice under homeostasis and in infected animals. Therefore, the Asm activity as well as sphingomyelin and ceramide concentrations were determined in the colon. As expected, we observed no Asm activity accompanied with a strong accumulation of sphingomyelin in non-infected and infected Asm KO mice and only slight changes in the ceramide concentrations (Figures 7A–C). To approve the physiological effect of Asm deficiency in comparison to amitriptyline mediated inhibition of Asm, we infected Asm WT and Asm KO mice via oral gavage with ~2 × 10^9^ CFUs *C. rodentium* per mouse and assessed the body weight, bacterial burden, spleen weight, colon weight-to-length ratio, and the histopathological score. Importantly, non-infected Asm WT mice and Asm KO mice exhibited no difference regarding the analyzed parameters (Figures 7D–F). In contrast, we observed an enhanced loss of body weight at day 10 post infection in Asm KO mice compared to Asm WT mice (Figure 7D). Determination of the bacterial burden in the feces showed that bacterial loads were higher in infected Asm KO mice on day 5 and 10 post infection compared to infected Asm WT mice (Figure 7G). In addition, a tendency of systemic dissemination in the liver and the spleen was observed in infected Asm KO mice but not in infected WT mice (Figures 7H,I). Well in line, also spleen weights and colon weight-to-length ratios were higher in infected Asm KO mice than in Asm WT mice (Figures 7E,F). Finally, the histopathological analysis of colon tissues revealed significantly elevated crypt hyperplasia and inflammatory scores in infected Asm KO mice compared to infected Asm WT mice (Figures 7J–L). In summary, Asm KO mice are strongly susceptible to *C. rodentium* infection and show the same histopathological phenotype as infected amitriptyline treated animals.

## Discussion

The gastrointestinal tract is the largest mucosal surface in the human body, fulfilling the pivotal role of nutrition and water absorption. Pathogens preferentially invade the host through the gastrointestinal tract forcing it to distinguish between harmless and beneficial bacteria. Thus, the gastrointestinal tract adapted to these unique circumstances by limiting direct bacterial contact to the epithelial cell surface, rapid detection and killing of invading bacteria, and minimizing the exposure of commensal bacteria to the immune system (41). Dysregulation of this uniquely balanced system can lead to chronic inflammation, resulting in IBD (42). Although the exact etiology of IBD remains unclear, the role of sphingolipids in contributing to the inflammatory process is evident (4, 5, 43–47). Asm is of particular interest, as it catalyzes the hydrolysis of sphingomyelin to ceramide, which is the central molecule in the sphingolipid metabolism. Thus, research on therapeutic agents able to modulate Asm and tissue-specific delivery systems or application routes is mandatory (47).

The importance of Asm was first recognized in Niemann-Pick disease type A and B, also called Acid Sphingomyelinase Deficiency. Thus, sphingomyelin cannot be metabolized properly and is accumulated within cells, eventually causing cell death and the malfunction of major organ systems (10). Of note, recent studies point out that patients with Niemann-Pick disease are susceptible to pathogen infections (14, 48), indicating the association between deficiency and aberrant immune responses. Well in line, in the present study we identified Asm as a critical regulator of mucosal immunity to the *C. rodentium*. Asm inhibition or Asm deficiency strongly enhanced the susceptibility to enteric *C. rodentium* infection.

Interestingly, the impact of Asm and ceramide on IBD is intensively discussed. Evidence that Asm may be a therapeutic target in colitis has been demonstrated by a study using the Asm inhibitor SMA-7. SMA-7 inhibited LPS-induced activation of NFκB and release of pro-inflammatory cytokines TNFα, IL-1β, and IL-6 in macrophages (12). This was correlated with decreased ceramide production. In a chemically induced mouse model of colitis, oral administration of SMA-7 resulted in decreased cytokine levels in the colon and lower severity of colonic injury. Meanwhile, also desipramine and amitriptyline treatment was shown to inhibit Asm and to decrease pathology in common acute and chronic epithelial injury colitis models (13, 49). In all three studies, Asm inhibition resulted in a decreased pro-inflammatory cytokine production and impaired lymphocyte infiltration. Importantly, broad immunosuppression increases the risk of infectious complications (17) and several enteropathogens have been implicated in the development of gastrointestinal diseases (50, 51). Infection with *Salmonella (S.) typhimurium* results in severe gastroenteritis (52, 53). The pathogen infects the host by invading macrophages in Peyer's patches. Intracellular *S. typhimurium* survives within the lysosomal compartment by preventing lysosomal maturation of the phagosomes (54). Asm activity is required for the release of the reactive oxygen species that are necessary for the killing of intracellular *S. typhimurium* (55). Therefore, Asm deficiency strongly enhance the susceptible to *S. typhimurium* infection (56). Although we also observed a strong susceptibility of Asm inhibited and Asm deficient mice to *C. rodentium* infection, we could not observe any changes in the frequency and phenotype of macrophages in infected amitriptyline treated mice compared to infected wild type mice. However, *C. rodentium* is a noninvasive, attaching-effacing enteric bacterial pathogen, which does not infect macrophages but directly interacts with the intestinal epithelial layer (57). Therefore, it is more likely that the intestinal barrier is impaired in amitriptyline treated animals, which is well in line with the enhanced systemic bacterial distribution of *C. rodentium* in amitriptyline treated mice. Indeed, intestinal sphingolipids provide a non-specific barrier. In a porcine model, inhibition of *de novo* ceramide synthesis impaired the proliferation and barrier function of intestinal epithelial cells, which in turn led to the induction of inflammation (45). Furthermore, intestinal deletion of serine palmitoyltransferase (SPT), which is the rate-limiting enzyme for sphingolipid biosynthesis, significantly decreased the ceramide and sphingomyelin levels in the plasma membrane of gut cells and promoted intestinal cell apoptosis with the impairment of gut barrier function (58). However, further investigation is required to fully understand the impact of Asm on the barrier function in infectious and non-infectious colitis.

T_h_1 and T_h_17 cells, as part of the adaptive immunity, mediate the host defense against *C. rodentium* via the production of their signature cytokines IFNγ and IL-17 (26, 59), and mice depleted of either cell type have an impaired ability to clear the infection (29, 60). However, an uncontrolled CD4^+^ T cell response leads to severe immunopathology (20). Interestingly, we observed a significant increase of T_h_1 and T_h_17 cells accompanied with severe immunopathology in infected mice with Asm inhibition compared to infected wild type mice. Well in line, the regulation of murine T_h_1 differentiation by ceramide has been reported. A ceramide analog was shown to enhance IL-12 induced T_h_1 differentiation with increased T-bet expression and IFNγ production (61). In contrast, Asm inhibition in human CD4^+^ T cells abrogates T_h_1 cell differentiation (62). In addition, inhibition of Asm bioactivity by imipramine, which blocks the generation of ceramide, was shown to impair T_h_17 generation by blocking both mTor and Stat3 (63). Interestingly, we showed an enhanced frequency of T_h_17 cells in amitriptyline treated infected mice. Of note, amitriptyline also inhibits in part acid ceramidase, which metabolizes ceramide to sphingosine. Consequently, infected Asm inhibited mice displayed increased ceramide concentrations in the colon compared to infected wild type mice, which seems to further boost the T_h_17 response. During *C. rodentium* infection, T_h_1 and T_h_17 immunity is counterbalanced by regulatory T cells to inhibit severe immunopathology. Asm was recently described as negative regulator of T_reg_ development (64). In comparison to wild type mice, Asm deficient mice have a higher number of systemic T_reg_ cells under homeostasis. Furthermore, inhibition of Asm and supplementation of IL-2 result in augmentation of Foxp3 expression as well as induction of T_reg_ differentiation *in vitro* (64, 65). Surprisingly, we identified lower frequencies of T_regs_ in the colon of infected Asm inhibited mice compared to infected wild type mice suggesting differences in the polarization process under homeostasis and during infection. Finally, in most of the *in vivo* studies the Asm inhibitor was applied intraperitoneally (66–68), whereas we chose the oral route via the drinking water to specifically target the gastrointestinal tract. Hence, we cannot exclude that the specific immunosuppressive micro-milieu in the intestine interferes with the effects induced by Asm inhibition.

In summary, in contrast to the protective effect of Asm inhibition in common acute and chronic epithelial injury colitis models, Asm inhibition or Asm deficiency strongly enhanced the susceptibility to enteric *C. rodentium* infection. Therefore, understanding the sphingolipid enzymes and metabolic pathways involved in regulating intestinal inflammation under infectious and non-infectious conditions is the prerequisite for the development of new therapeutic strategies, which target the sphingolipid metabolism.

## Data Availability

All datasets generated for this study are included in the manuscript and/or the supplementary files.

## Ethics Statement

This study was carried out in accordance with the recommendations of the Society for Laboratory Animal Science (GV-SOLAS) and the European Health Law of the Federation of Laboratory Animal Science Associations (FELASA). The protocol was approved by the North Rhine-Westphalia State Agency for Nature, Environment and Consumer Protection (LANUF), Germany.

## Author Contributions

JM and AW: conceived and designed the experiments and wrote the paper. JM, VP, RK, J-FE, FS, LJ, AY, KB, and JZ: performed the experiments. JM, VP, RK, J-FE, LJ, WH, and AW: analyzed the data and reviewed and edited the manuscript. MH, DH, BK, RNK, JB, and WH: contributed reagents, materials, analysis tools.

### Conflict of Interest Statement

The authors declare that the research was conducted in the absence of any commercial or financial relationships that could be construed as a potential conflict of interest.
